# Noise Analysis of Monolayer Graphene Nanopores

**DOI:** 10.3390/ijms19092639

**Published:** 2018-09-06

**Authors:** Zi-Yin Zhang, Yun-Sheng Deng, Hai-Bing Tian, Han Yan, Hong-Liang Cui, De-Qiang Wang

**Affiliations:** 1 College of Instrumentation and Electrical Engineering, Jilin University, Changchun 130061, China; zhangziyin@cigit.ac.cn; 2 Chongqing Institute of Green and Intelligent Technology, Chinese Academy of Sciences, Chongqing 400714, China; dengys@sustc.edu.cn (Y.-S.D.); tianhaibing@cigit.ac.cn (H.-B.T.); yanhan@cigit.ac.cn (H.Y.); 3 Materials Characterization and Preparation Center, Southern University of Science and Technology, Shenzhen 518055, China

**Keywords:** monolayer graphene, solid-state nanopore, suspended area, noise, power spectral density

## Abstract

Graphene-based nanopore devices have shown tantalizing potential in single molecule detection for their monoatomic membrane thickness which is roughly equal to the gap between nucleobases. However, high noise level hampers applications of graphene nanopore sensors, especially at low frequencies. In this article, we report on a study of the contribution of suspended graphene area to noise level in full frequency band. Monolayer graphene films are transferred onto SiN*_x_* substrates preset with holes in varied diameters and formed self-supported films. After that, the films are perforated with smaller, nanoscale holes. Experimental studies indicate a dependency of low-frequency 1/*f* noise on the underlying SiN*_x_* geometry. The contribution of the suspended graphene area to capacitance which affects the noise level in the high frequency range reveals that the graphene free-standing film area influences noise level over a wide frequency region. In addition, the low-frequency noise demonstrates a weak dependency on salt concentration, in deviation from Hooge’s relation. These findings and attendant analysis provide a systematic understanding of the noise characteristics and can serve as a guide to designing free-standing monolayer graphene nanopore devices.

## 1. Introduction

Solid-state nanopores are uniquely suited for single molecule detection due to their superior attributes over biological nanopores that allow long service period, specific design towards concerted detection target and compatibility with devices [1,2,3]. Different from conventional long-channel nanopores, graphene is one of the typical 2D materials whose layer thickness is 0.34 nm, to the extent that its sub-nanometer scale approaches the interval between two contiguous bases in a single DNA molecule. This characteristic herald the high spatial resolution of graphene nanopores profited from its sensitivity to the small change of the molecule passing through the nanopore. Furthermore, compared to commonly insulated membrane materials used for nanopore sensor fabrication such as silicon nitride (SiN*_x_*) [4], aluminum oxide (Al_2_O_3_) [5], and silicon oxide (SiO_2_) [6], graphene has good electronic conductivity and robust mechanical properties. Despite these superiorities, high noise levels impede the applications of graphene nanopore sensors. 

Indeed, the reduction of noise in graphene nanopore sensor is the most intractable problem. In allusion to that, several studies have been implemented, such as utilizing multiple layers of graphene [7,8], deposition of titanium dioxide (TiO_2_) [8], alumina [9] or polyimide [10] over graphene and fabrication of an Al_2_O_3_/graphene stack structure [11]. However, such structural transformations of devices increase membrane thickness. Garaj’s work [12] demonstrates that the current density intensively distributes near the wall inside of an atomically short nanopore, but is nearly homogeneous in long channel nanopores like SiN*_x_* when applying a bias voltage on both sides of the membrane, which produces sub-nanometer scale sensitivity. Therefore, the original expectation of single-nucleobase resolution will be sacrificed as the result of any thickness increase during the modification process.

Under the premise of holding the line on single-atomic thickness, one simple and effective method of depositing SiN*_x_* layer beneath the graphene had been put forward to cope with these issues. The underlying SiN*_x_* membrane is more than a supporter to offer mechanical stabilities, but also mitigates pin-hole effect as well. Schneider [13] and his colleagues drilled a nano-size hole in graphene flakes placed over a silicon nitride membrane with a micro-size aperture, resulting in noise reduction. Garaj et al. [12] fabricated and measured two graphene nanopores suspended over 200 × 200 nm and 20 × 20 nm apertures respectively in thin and free-standing SiN*_x_* films supported by silicon substrates, from which they found it was apparently feasible to reduce the noise level of graphene by reducing the suspended area; although this work was simply verified by comparing individual current traces, it indicated an underlying reason of mechanical fluctuation contributing to the high noise level.

Noise characteristic polynomial is beneficial for the improvement of experimental program to minimize noise level as much as possible and optimize the signal-to-noise ratio in single molecule translocation experiment. Noise in a solid-state nanopore consists of four components and can be described by the noise characteristics:

(1) $$S=Af^{-\beta }+B+Cf+Df^{2}$$

where *f* is frequency, and *β* is a fitting parameter (β≈1). And the parameters A, B, C and D in the four terms represent 1/*f*, thermal, dielectric and amplifier noise respectively [10]. The noise of the graphene nanopore scheme in full spectral band is usually divided into two parts, a low-frequency regime and a high-frequency regime [14]. At low-frequency, noise power is primarily relevant to 1/*f* [9], while the high-frequency noise in solid-state nanopores is dominated by the effective capacitance of the devices [15]. The effective capacitance consists of the summation of capacitances of the membrane and other parasitic capacitances generated by the substrate and the Debye layer [15]. The employment of high quality substrate material substantially reduces the effects of parasitic and overall capacitance, and leads to improvement of dielectric noise. Low-frequency noise with a dependence of current spectrum density on 1/*f* is a ubiquitous phenomenon plaguing many electronic systems. Over the last decades, some studies have been conducted to explore the mechanism of 1/*f* noise generation. However, the origin and mechanism for suppression of low-frequency noise of solid-state nanopores remain elusive. Hooge’s model proposed that the low-frequency noise has nothing to do with surface effect but only with fluctuation in charge carriers [16], while Balandin meliorated the model and took the surface charge into consideration [17]. The 1/*f* noise is governed by the volume noise when the membrane thickness exceeds seven atomic layers (about 2.5 nm), which means that the fluctuation of current and surface charge contribute to the 1/*f* noise below this thickness [18].

Here, we discuss the influence of free-standing area of graphene membrane on low-frequency noise level. We carried out a systematic study from which we concluded that the mechanical fluctuation of monolayer graphene membrane contributes to the low-frequency 1/*f* noise significantly. In addition, resistance and capacitance of the membrane are corroborated to be associative with suspended area of graphene as well, which are in vital connection with noise power magnitude in the low and high frequency ranges separately. Moreover, deviating from Hooge’s relation, the 1/*f* noise shows a weaker dependency on electrolyte concentration, and surface charges are found to impact on pore conductance fluctuation in graphene nanopores.

## 2. Results

To investigate the dependency of noise power on mechanical membrane fluctuation, we fabricated 8 graphene nanopores with similar diameters over a series of larger silicon nitride pores. The free-standing film area of graphene film, named suspended area, is equivalent to the size of SiN*_x_* pore. Table 1 shows the geometric setting of all nanopores fabricated.

### 2.1. Low-Frequency 1/f Noise in Single-Layer Graphene Nanopore Devices

**Figure 1:**
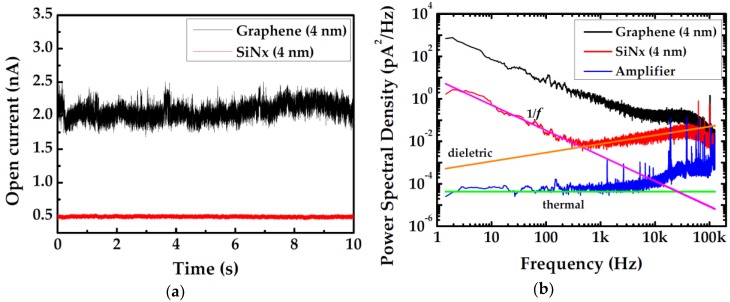
Comparison of noise level in different material nanopore systems measured at 1 M KCl and 100 mV: (**a**) Open current of two comparable nanopores punched on graphene and SiN*_x_*, respectively; and (**b**) noise power spectral densities of the two nanopores. The fitting executed according to the noise polynomial $S=Af^{-\beta }+B+Cf+Df^{2}\;$ is shown for the SiN*_x_*-based nanopore sensor.

Figure 1 depicts the noise levels of two nanopores with similar diameters which are drilled on different materials. All data are measured at 1 M KCl solution and 100 mV bias voltage. Figure 1a shows the open current traces of the two nanopores, from which it can be obviously observed that the open current of graphene nanopore appears less steady than SiN*_x_*. Figure 1b is the noise power spectrum obtained by conducting Fourier transformation on the time-domain current response. The inherent noise of amplifier is measured by recording the open current in air without any sample or solution. The magenta, orange and blue are straight lines fit to the low-frequency 1/*f* noise, high-frequency dielectric noise and thermal noise, respectively.

**Figure 2:**
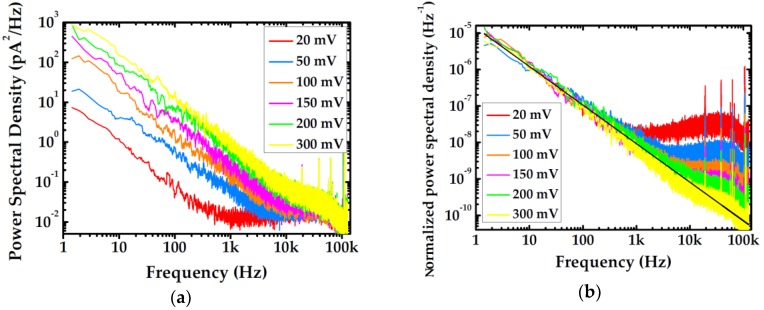
Noise traces of a graphene nanopore with diameter of 5 nm at 100 mM KCl: (**a**) Noise power spectral density at varied bias voltage; and (**b**) normalized noise power spectral densities. The black straight line represents the average result of the low-frequency linear fitting.

After Fourier transform, the noise power spectra of 8 graphene nanopores at varied KCl concentrations and bias voltages are obtained. The noise power spectral density of a graphene nanopore (No. 5) at 100 mM KCl is shown in Figure 2a for instance. The noise power (A) is a function of inverse frequency according to the Hooge relationship [16]:

(2) $$\frac{S_{I}}{I^{2}}=\frac{A}{f}=\frac{\alpha_{H}}{N_{c}f^{\beta }}$$

where $\alpha_{H}$ and $N_{c}$ are Hooge parameters and corresponding charge carrier number respectively. By dividing the noise power spectral density by the corresponding square value of average current, the noise power spectrum is normalized ($S/I^{2}$), and at frequency equal to 1 Hz the normalized power spectrum ($S/I^{2}$) is intercepted at A, thus the intercept of linearly fitted normalized low-frequency noise determines the magnitude of low-frequency noise power (A) as shown in Figure 2b. In this work, the current noises are measured at bias voltages of 20, 50, 100, 150, 200, and 300 mV. After the normalization process, changes of low-frequency noises at different bias voltages exhibit no conspicuous variation and therefore the linear fit of 1/*f* noise displayed in Figure 2b can be considered as the average of the results at six applied voltages. All low-frequency noise data at various concentration are obtained by this means. Confirmed by Kumar [15], the noise power almost remains invariant as the open current of nanopore increases, which means nanopore current does not promote noise power but simply makes it more prominent. Considering the fluctuation of conductance, the instability is probably caused by the change of effective diameter which can be induced by incomplete shedding, reblocking, and mechanical fluctuation of graphene membrane, and the average operation can also serve to assess experimental error.

**Figure 3:**
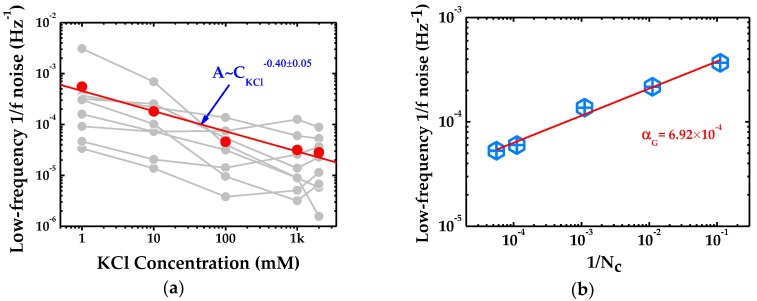
Verification of Hooge’s relation in single-layer graphene based nanopore system: (**a**) Dependency of low-frequency noise on KCl concentration. The red points are the average of eight pores at varied KCl concentration. Likewise, the red curve is the linear fitting of the mean values at different KCl concentration; and (**b**) the relationship between low-frequency noise and inverse number of charge carriers. The fit line determines the noise coefficient ($\alpha_{G}$) in the graphene nanopore of No. 2.

Figure 3a depicts low-frequency noise power (A) as a function of KCl concentration ($C_{\mathrm{KCl}}$). The noise power of individual pore at varied salt concentrations does not seem to follow a linear trend strictly for the possibility of the change of equivalent diameter and current fluctuation in monolayer graphene. The fit result of the mean values (red points) coming from the 8 graphene samples demonstrates that the data yield 0.40±0.05. Since the relationship between noise power (A) and its coefficient ($\alpha_{G}$) can be described as $A=\frac{\alpha_{G}}{N_{c}}$, the value of noise coefficient in the nanopore of No. 2 can be calculated as illustrated in Figure 3b, where the estimated value of $\alpha_{G}$ is found to be $6.92\times 10^{-4}$.

**Figure 4:**
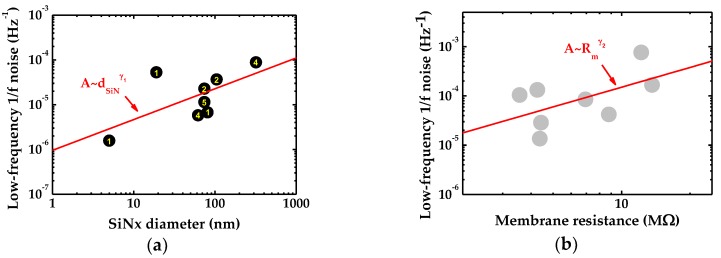
(**a**) Low-frequency noise power plotted against the underlying SiN*_x_* diameter. The yellow number in the center of the points represent the pore size of each graphene nanopore; and (**b**) the noise in the low-frequency range versus membrane resistance. The red solid line fits to the resistance of eight graphene nanopores.

To investigate the influence caused by membrane fluctuation on low-frequency 1/*f* noise, the low-frequency noises (A) of eight graphene nanopores measured at 2 M KCl are plotted against the diameter of underlying SiN*_x_* ($d_{SiN_{x}}$) in Figure 4a. The result of linear fit in logarithmic space displays the dependency of $A\sim d_{SiN_{x}}{}^{\gamma_{1}}$, where the $\gamma_{1}=0.69\pm 0.34$ and we find that the diameter of the underlying SiN*_x_* film strongly impacts on the low-frequency noise of graphene nanopore sensors. Figure 4b shows the averaged low-frequency noise fitted to membrane resistance which yields a dependency of $\gamma_{2}=1.33\pm 0.80$.

### 2.2. Membrane Test and Parameters Analysis

**Figure 5:**
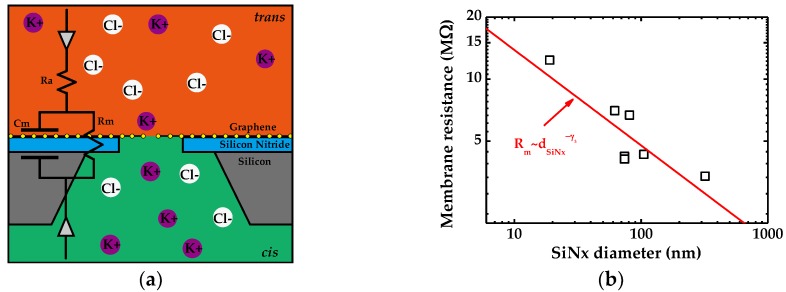
(**a**) Schematic of free-standing monolayer graphene nanopore and equivalent circuit of the system; and (**b**) the membrane resistance as a function of underlying SiN*_x_* diameter.

According to the equivalent circuit model of a generic graphene nanopore device, the resistance and capacitance of the membrane contribute to system noise significantly. In this work, we conducted a membrane test experiment on individual pores. The equivalent circuit diagram of the nanopore system is shown in Figure 5a, and Figure 5b depicts the membrane resistance of seven nanopores (No. 2–No. 8) as a function of SiN*_x_* diameter at 2 M KCl. The red solid line in this graph displays the fit product $R_{m}\sim d_{SiN_{x}}{}^{-\gamma_{3}}$ in which $\gamma_{3}=0.46\pm 0.14$.

**Figure 6:**
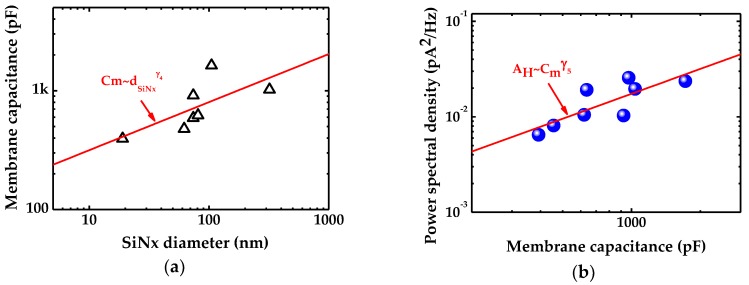
Impact of graphene suspended area on noise in high-frequency region: (**a**) Membrane capacitance measured by membrane test in 2 M KCl solution versus underlying SiN*_x_* diameter; and (**b**) the relationship between high-frequency noise and membrane capacitance. The fit line determines a dependency of high-frequency noise on membrane capacitance.

The capacitance at 2 M KCl solution against SiN*_x_* diameter is shown in Figure 6a and a linear fit in logarithmic space yields a dependency of $\gamma_{4}=0.40\pm 0.19$. Capacitance involved refers to effective capacitance including capacitance arisen from all parasitic elements. The chip of sample No. 1 ($d_{SiN_{x}}=5\;\mathrm{nm},\;d_{G}=1\;\mathrm{nm}$) is influenced by its graphene hole dramatically so that it exhibits large error in linear fit to SiN*_x_* diameter. Thus, it is neglected in Figure 6a. Figure 6b depicts noise in high frequency as a function of membrane capacitance with a relationship of $A_{H}\sim C_{m}{}^{\gamma_{5}}$ where $\gamma_{5}=0.87\pm 0.27$.

**Figure 7:**
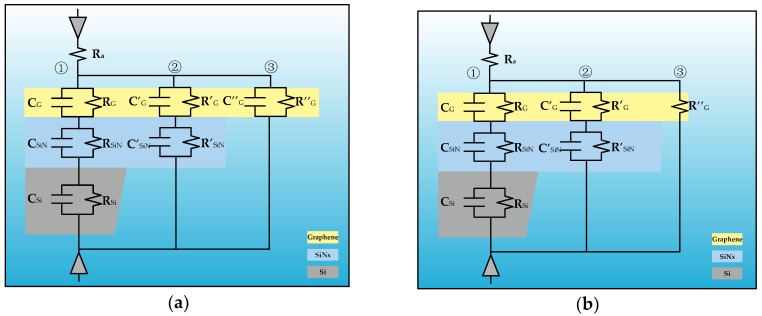
Equivalent circuit of monolayer graphene nanopore regime: (**a**) equivalent circuit of the extensive suspended model; and (**b**) the equivalent circuit of the narrow free-standing model.

An equivalent circuit model is conducive to investigation of noise characteristic in nanopore system. The universal equivalent circuits are plotted in Figure 7. A graphene nanopore can be regarded as an equivalent circuit consisting of three branches numbered 1, 2, and 3. C/R, C′/R′, and C″/R″ represent the capacitance and resistance in each corresponding branch, respectively. Under the condition of extensively suspended graphene, the equivalent circuit of monolayer graphene nanopore can be illustrated as Figure 7a, in which each color represents a corresponding membrane layer.

However, the theoretical model is quite distinct when there is not much difference between the two apertures of graphene and SiN*_x_*. Under this condition, the free-standing part (part 3) of graphene membrane diminishes drastically and its equivalent capacitor can be omitted. The equivalent circuit should be represented by Figure 7b. The nanopore system serves as a long and narrow channel as the the pore size and suspended area of graphene are at the same order of magnitude. This is exactly the reason for the qualitative difference of sample No. 1 from the rest of the samples.

From experimental observation, the inversion frequency that divides the full band into a low-frequency range and the high-frequency range is found to be variable. A typical inversion frequency at 1 M KCl solution and 150 mV bias voltage ranges from 300 to 20 kHz while the aperture of underlying SiN*_x_* lies in the range of 5–320 nm.

### 2.3. Ionic Behavior in Single-Layer Graphene Nanopore

**Figure 8:**
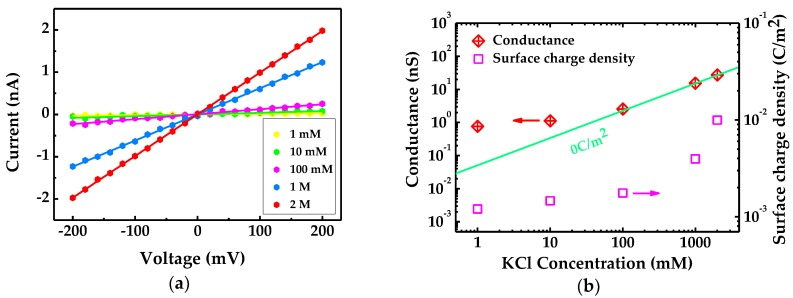
(**a**) *I*-*V* curve of a graphene nanopore with a diameter of 1.2 nm; and (**b**) the conductance and surface charge density against concentration of KCl. The green line shows the fitting without contribution of surface charge distribution.

Figure 8a illustrates ionic current versus voltage (*I*-*V*) characteristic of single-layer graphene nanopore with diameter of 1.2 nm at varied KCl concentration. The calculated slopes of the solid lines fitting to the dots represent the conductance of individual nanopores. The estimated conductance of 8 fabricated graphene nanopores lies in the range of 4–39 nS. The conductance and surface charge density as functions of electrolyte concentration are shown in Figure 8b. The green line represents the calculated conductance without surface charge effect involved.

## 3. Discussion

Graphene is the most widely used 2D material which shows great prospect of future success in realization of single-base spatial resolution in DNA sequencing. However, as shown in Figure 1, graphene nanopore demonstrates higher noise level than SiN*_x_*-based device. It can be obviously observed in Figure 1a that the current trace of a 4 nm graphene nanopore is four times higher than that of a 4 nm SiN*_x_* nanopore. Additionally, the current fluctuates much more dramatically than the current of the SiN*_x_* nanopore, which exhibits a significant low-frequency variation. As a matter of fact, the disparity of noise levels in different materials appears more prominent in the noise power spectrum. From Figure 1b, it can be found that the low-frequency 1/*f* noise of graphene is more than two orders of magnitude higher than that of SiN*_x_*. Meanwhile, current noise in high-frequency seems disparate as well, even though the substrate materials are identical. Normally, a demarcation frequency separates the full spectrum band into low-frequency and high-frequency regions. A previous work has proposed that it lies at about 1 kHz, below which the 1/*f* noise would govern the noise power of the whole system [14]. In this work, however, it is found to be a variable that changes with voltage, electrolyte concentration, pore size, free-standing area and even material. However, bias voltage does not contribute to low-frequency noise power which can be ascertained from Figure 2. Power spectral density manifests a wider low-frequency band when a higher bias voltage is applied, but 1/*f* noise ceases to dominate beyond 10 kHz. Calculated 1/*f* noise power after normalization is nearly equal, which further proves the applied voltage’s irrelevancy with 1/*f* noise power.

In Hooge’s relation, noise of 1/*f* regime shows a strong dependency on electrolyte concentration ($A\sim C_{\mathrm{KCl}}{}^{-1}$). To assess the general applicability of Hooge’s relation in monolayer graphene system, the low-frequency noise power at various pore sizes and salt concentration are plotted in Figure 3a. However, linear fit of the mean noise power only reveals a weak dependency on electrolyte concentration ($A\sim C_{KCl}{}^{-0.40\pm 0.05}$) and the computed noise coefficient $\alpha_{G}=6.92\times 10^{-4}$ for monolayer graphene nanopore (No.2) shown in Figure 3b exhibits a value slightly higher than those of SiN*_x_* [19] and few-layer graphene [15].

Next, we inspect the influence caused by membrane fluctuation on low-frequency 1/*f* noise. In Figure 4a, the noise power A of the 8 graphene nanopores ranges from $1.6\times 10^{-6}$ to $8.8\times 10^{-5}$. The samples of No.3–No.6 have graphene nanopore diameter of 4 nm, 2 nm, 5 nm and 1 nm respectively suspended over similar SiN*_x_* holes, of which the low-frequency noise ranges from $5.8\times 10^{-6}$ to $2.3\times 10^{-5}$. Indeed, the diameter of graphene pore $d_{G}$ has an influence on the 1/*f* noise, albeit not significant compared to that of $d_{SiN_{x}}\;$ and is negligibly small in the present work. Most of the data points follows a generally linear trend and illustrates a dependency of $A\sim d_{SiN_{x}}{}^{\gamma_{1}}$ with $\gamma_{1}=0.69\pm 0.34$, which gives an evidence to address the contribution of underlying SiN*_x_* geometry, videlicet membrane fluctuation contributes to 1/*f* noise.

To pin down the origin of noise, it is heuristic to provide the equivalent circuit model of solid-state nanopore. The structure schematic of monolayer graphene nanopore sensor is shown in Figure 5a and it can be regarded as a resistor in parallel with a capacitor resulting from the liquid contact to the silicon chip containing the nanopore. Utilizing membrane test function of a patch clamp amplifier, we measured the resistance and capacitance of the drilled graphene membrane. Based on membrane resistance, the 1/*f* noise demonstrates a dependency of $A\sim R_{m}{}^{\gamma_{2}}$ with $\gamma_{2}=1.33\pm 0.80,$ showing a remarkable agreement with Heerema’s work (1.4±0.4), and a discrepancy from Hooge’s relation which considers $\gamma_{2}=3$ instead [14]. The relations between membrane resistance and capacitance against diameter of underlying silicon nitride pore are plotted in Figure 5b and Figure 6a separately. Linear fits in logarithmic space reveal their dependencies on suspended area of $R_{m}\sim d_{SiN_{x}}{}^{-0.46\pm 0.14}$ and $C_{m}\sim d_{SiN_{x}}{}^{-0.40\pm 0.19}$. For silicon nitride pores the resistance can be modeled as:

(3) $$R=\sigma^{-1}\left(\frac{4L}{\pi d^{2}}+\frac{1}{d}\right)$$

where σ, *L* and *d* stand for conductivity of salt concentration, channel length and pore diameter. The two terms represent pore channel resistance and access resistance respectively. Since the graphene is extremely thin, previously reported works suggest that the channel resistance can be ignored and in monolayer graphene nanopores the total resistance can be described as: $R_{G}=\sigma^{-1}\frac{1}{d_{G}}$ [14]. However, the experimental results indicate a dependency of membrane resistance on the underlying SiN*_x_* pore size and therefore contribution of graphene’s suspended area cannot be neglected. It can be found in Figure 5b that the resistances of No.4 and No.5 show indistinctive difference (4.2 and 4.1 MΩ respectively), corresponding to graphene nanopore diameter of 2 and 5 nm, respectively, suspended over SiN*_x_* holes with the same size of 74 nm. This implies that what contributes to the analysis of resistance significantly is not the diameter of graphene nanopores, rather it is the suspended graphene area in the present work. Basically, the influence of the variation of the graphene pore size on the analysis of suspended-area-related resistance would be negligibly small in this case.

As aforementioned, current noise in high-frequency range is affected by membrane capacitance. Electrolyte solution in the cis and trans chambers constitute the two plates of a parallel-plate capacitor while the membrane serves as the medium. Previous work on high-frequency noise suggests that it scales with nanopore capacitance as [19]:

(4) $$S_{D}=4k_{B}\mathrm{TCD}(2\pi f)$$

where $k_{B}$, T, C, and D represent Boltzmann constant, absolute temperature, effective capacitance and loss tangent respectively. To access this relation, noise at 2 M KCl solution and 1 kHz is delineated in Figure 6b versus membrane capacitance. Observed from Figure 2a, the amplitude of current noise in high frequency is impervious to variations of bias voltage, so the applied high-frequency noise is calculated from current trace at 0 mV. The affiliation of membrane capacitance to high-frequency noise exhibits $A_{H}\sim C_{m}{}^{\gamma_{5}}$ and $\gamma_{5}=0.87\pm 0.27$ which basically conforms to accepted noise theory.

Conductance of all graphene chips range from 4 to 39 nS, which, compared to previously reported studies [20], attests to the high quality of the single-layer graphene films we used. Conductance in graphene nanopore system involving surface charge effect follows the relation [19,21,22]:

(5) $$G=\sigma^{\prime }{\left[\frac{4L_{G}}{\pi d_{G}{}^{2}}+\frac{1}{d_{G}}\right]}^{-1}+\mu_{K^{+}}\sigma \frac{\pi d_{G}}{L_{G}}$$

where σ′, $\mu_{K^{+}}$ and σ reflect the values of solution conductivity, potassium ion mobility, and surface charge density separately, with $\sigma^{\prime }=eN_{\mathrm{KCl}}\left(\mu_{K^{+}}+\mu_{{\mathrm{Cl}}^{-}}\right)$. At low electrolyte concentration, surface charge fluctuates against solution concentration and contributes to nanopore conductance, and then becomes innocuous as the concentration rises further.

## 4. Materials and Methods

### 4.1. Sample Preparation

As the schematic of the devices illustrated in Figure 5a shows, we utilized commercially purchased SiN*_x_* chips (Norcada, Edmonton, AB, Canada) as substrates of graphene membranes. The 20 nm-thick low-stress SiN*_x_* membrane is freestanding over a silicon flake with a 20 × 20 μm window at its center. After N_2_ plasma cleaning for 2 min, holes of diameters ranging from 5 to 320 nm were drilled and measured through the SiN*_x_* membrane with focused ion beam (FIB, Carl Zeiss, Orion NanoFab, Peabody, MA, USA) [23,24].

### 4.2. Graphene Transference

The graphene we used is produced with chemical vapor deposition method on copper sheet and a layer of polymethyl methacrylate (PMMA) was spin-coated on it. After 20 min heating, the PMMA film formed and then was immersed into acid solution consisting of HCl:H_2_O_2_:H_2_O = 1:1:10 to remove the copper. Subsequently, the PMMA film with graphene on it was covered onto the fabricated SiN*_x_* chips. Finally, the chips were drowned in acetone to wash the PMMA off.

### 4.3. Graphene Nanopore Fabrication and Measurement

The graphene nanopores were drilled by dielectric breakdown [25]. In advance of the dielectric breakdown process, the graphene device was soaked in EtOH for 30 min to obliterate organic residue. Thenceforth, the chip was embedded into a customized fluidic cell which was made of PDMS and then a buffer solution was introduced to cover both sides of the fluidic cell. The graphene drilling and subsequent measurement were executed in the buffered environment composed of potassium chloride, 10 mM Tris-HCl and pH 8.0, where the Ag/AgCl electrodes were immersed and connected to the controlled breakdown equipment and patch clamp amplifier system respectively. The dielectric breakdown works as a step growth of electrical pulse. The pulse drives the ions inside the electrolyte to strike the graphene membrane and gradually form a tiny defect. Finally, the defect turns into a nanoscale hole [26,27,28]. Owing to the thinner membrane of monolayer graphene, the processing time was shorter than that of SiN*_x_* reported [25], and the sudden drops of measured voltage took place when the current stimulation was around 30 nA, which represented the formation of a single-nanopore. All the data measurement discussed here were conducted with an Axopatch 200B (Molecular Devices; Sunnyvale, CA, USA) amplifier digitized at 250 kHz with a 100 kHz six-pole Bessel filter.

### 4.4. Statistical Analysis

Power spectral densities were calculated by taking the Fourier transformation of every open current in diverse applied voltage. Then the power spectral density was divided by the squared value of the mean current that was worked out through Gaussian fitting, which revealed the normalization of noise power density. Under general conditions, the low-frequency 1/*f* noise contributes in a finite frequency region and the limiting frequency, as we have observed, varies from 300 Hz to 20 kHz, perhaps even higher depending on values of the bias voltage, solution concentration, suspended area and diameter of graphene nanopore.

## 5. Conclusions

In summary, we have provided an elaborate description and detailed analysis about the impact caused by the suspended area of monolayer graphene film to noise characteristic in a nanopore system. In the low-frequency region, noise with a dependency on the reciprocal frequency does originate from mechanical membrane fluctuation following a relation of $A\sim d_{SiN_{x}}{}^{0.69\pm 0.34}$. On the other hand, previously reported studies proposed that nanopore resistance depended only on pore size, which is different from the observation of this work ($R_{m}\sim d_{SiN_{x}}{}^{-0.46\pm 0.14}$). That low-frequency 1/*f* noise should scale with resistance according to Hooge’s relation with a power law of $A\sim R^{3}$ is in discrepancy from our experimental observation of $A\sim R^{1.33\pm 0.80}$. Furthermore, dependency of 1/*f* noise on charge carrier density $A\sim C_{KCl}{}^{0.40\pm 0.05}$ is found to be weaker in monolayer graphene device.

At high frequencies, noise power is related to nanopore capacitance dominated by its suspended area as well. Capacitances of 8 fabricated monolayer graphene nanopores are measured by utilizing membrane test and the results suggest a dependency on underlying SiN*_x_* pore geometry. The fit of noise versus membrane capacitance ($A_{H}\sim C_{m}{}^{0.87\pm 0.27}$) demonstrates consistency to commonly accepted noise model in the high-frequency regime.

In addition, we also discuss ionic behavior of monolayer graphene nanopore devices in this article and show that measured conductance deviates dramatically from linear fit of complete bulk conductance at low salt concentration. It indicates that surface charges and their fluctuation contribute to conductance and its uncertainty significantly.

## Figures and Tables

**Table 1:** Geometric parameters of nanopores.

Diameter (nm)	No. 1	No. 2	No. 3	No. 4	No. 5	No. 6	No. 7	No. 8
Graphene	1	1	4	2	5	1	2	4
SiN*_x_*	5	19	62	74	74	81	105	320
